# Response of neural reward regions to food cues in autism spectrum disorders

**DOI:** 10.1186/1866-1955-4-9

**Published:** 2012-05-17

**Authors:** Carissa J Cascio, Jennifer H Foss-Feig, Jessica L Heacock, Cassandra R Newsom, Ronald L Cowan, Margaret M Benningfield, Baxter P Rogers, Aize Cao

**Affiliations:** 1 Department of Psychiatry, Vanderbilt University, 1601 23rd Ave South, Suite 3057, Nashville, TN 37212, USA; 2 Vanderbilt Kennedy Center, Nashville, USA; 3 Department of Psychology and Human Development, Vanderbilt University, Nashville, USA; 4 Department of Pediatrics, Vanderbilt University, Nashville, USA; 5 Department of Radiology and Radiological Sciences, Vanderbilt University, Nashville, USA; 6 Institute of Imaging Science, Vanderbilt University, Nashville, USA

## Abstract

**Background:**

One hypothesis for the social deficits that characterize autism spectrum disorders (ASD) is diminished neural reward response to social interaction and attachment. Prior research using established monetary reward paradigms as a test of non-social reward to compare with social reward may involve confounds in the ability of individuals with ASD to utilize symbolic representation of money and the abstraction required to interpret monetary gains. Thus, a useful addition to our understanding of neural reward circuitry in ASD includes a characterization of the neural response to primary rewards.

**Method:**

We asked 17 children with ASD and 18 children without ASD to abstain from eating for at least four hours before an MRI scan in which they viewed images of high-calorie foods. We assessed the neural reward network for increases in the blood oxygenation level dependent (BOLD) signal in response to the food images

**Results:**

We found very similar patterns of increased BOLD signal to these images in the two groups; both groups showed increased BOLD signal in the bilateral amygdala, as well as in the nucleus accumbens, orbitofrontal cortex, and insula. Direct group comparisons revealed that the ASD group showed a stronger response to food cues in bilateral insula along the anterior-posterior gradient and in the anterior cingulate cortex than the control group, whereas there were no neural reward regions that showed higher activation for controls than for ASD.

**Conclusion:**

These results suggest that neural response to primary rewards is not diminished but in fact shows an aberrant enhancement in children with ASD.

## Background

Autism spectrum disorders (ASD) are a group of neurodevelopmental disorders defined by substantial impairments in social interaction and communication, as well as patterns of rigid or repetitive behavior, with onset in the first three years of life [1]. Social impairment is a central feature of ASD and is a primary target for neurobehavioral experimental studies. Much of this work has been pursued in the context of investigating differences in social perception and cognition, including the brain's detection and encoding of social information, attention to social stimuli, face recognition, and discrimination of social cues such as facial expression or gaze direction (for review, see [2]). Considerably less attention has been given to investigating the neural basis of possible differences in social motivation in ASD.

It has been suggested, however, that the social impairments seen in ASD may result from aberrant limbic mediation of the reward that typically drives social interaction. The rewarding nature of social attachment and social interactions [3] has led to speculation that neural reward mechanisms that typically reinforce and perpetuate social behavior are either dampened in ASD or recruited by non-social stimuli such as the objects of circumscribed interests or idiosyncratic sensory stimuli to which individuals with ASD may show intense attraction. However, it remains to be tested whether the affective basis of social deficits in ASD reflects aversion or simply lack of motivation (Thompson, B.L., personal communication), which may then implicate distinct but overlapping limbic circuits for avoidance (fear, disgust) or approach (reward). If the latter, it is unknown whether diminished motivation in ASD is limited to the reward of social stimuli or is a more generalized trait [4-6].

The hedonic experience of pleasure depends on endogenous opioid signaling in the ventral tegmental area (VTA) of the brain [7], which sends dopaminergic projections to the nucleus accumbens (NAc). The role of the NAc is to mediate the performance or work involved in reward seeking and anticipation [7-10]. These subcortical areas project reciprocally to the ventromedial prefrontal cortex (VMPFC) and orbitofrontal cortex (OFC) [11], which form associations between the sensory features of the reward stimulus and its hedonic value [12] through inputs from sensory cortices of every sensory modality to OFC [13]. In addition, the VMPFC and OFC regions compute expected reward versus reward outcomes to shape future behavior (Grabenhorst and Rolls, [14]). The insula is important for monitoring and evaluating the impact of external stimuli on internal states [15,16] and the amygdala is involved in evaluating emotional stimuli for their novelty [17], affective significance [18,19], and biological or behavioral relevance [20,21]. Separate but overlapping circuits and neurotransmitter systems mediate the hedonic ('liking') and the anticipatory ('wanting, craving') experiences of reward [9,22-24].

Palatable food is a potent stimulus for the reward system [23,25-27], as are food cues such as images of food [28,29]. The neural reward network hemodynamic response to food images is tightly correlated with reward sensitivity [30], and increases with the caloric content of the foods pictured [31] and with hunger motivational state (fasting versus satiated) [32]. Goldstone *et al*. [33]) noted an interaction between these two variables, such that the heightened response to high calorie versus low calorie foods was greater when fasting and concluded that hunger biases the neural reward system toward high calorie foods. Behavioral evidence corroborates this, as healthy adults under fasting conditions exhibit increased gaze duration to food images [34] and increased attentional capture by food images, resulting in decreased performance on a target detection task despite monetary incentives for accuracy [35]. These studies converge to suggest that images of high-calorie, palatable foods under fasting conditions constitute an effective stimulus that elicits response from neural reward networks.

Studies of the neural basis of reward in ASD have focused on contrasting social versus non-social (monetary) rewards, which have been found to have highly overlapping neural substrates [36]. Studies comparing ASD to typical control groups largely find diminished response to both social and monetary rewards [37,38]. Scott-Van Zeeland and colleagues noted significantly diminished response of the ventral striatum, anterior cingulate, and ventral prefrontal cortex, especially for social reward. Reported differences are generally stronger for social rewards [37,39] than for monetary ones. Using only monetary reward, without a contrast to social reward, Schmitz *et al*. [40] demonstrated an elevated blood oxygenation level dependent (BOLD) signal in the anterior cingulate in response to reward feedback in ASD. These discrepant results could be influenced by several variables that differed between studies, including the developmental stage (children versus adults) of the participants.

Although monetary reward paradigms are well established in their ability to recruit reward circuitry in typical adults, they may not be as ideal for individuals with ASD, who often do not manage their own money [41,42] and may have differences in abstract or symbolic representation even at the higher end of the spectrum [43]. If this is the case, it may be that the 'generalized' reward system differences seen in these studies were due to the choice of non-social reward, rather than a truly generalized deficit in reward system functioning in ASD.

A recent study by Dichter *et al*. [44] provides more information with which to address the question of alternative reward stimuli in ASD by contrasting monetary rewards with non-social objects as rewards. In this study, objects were selected to have a high likelihood of representing restricted interests in ASD (that is, images related to commonly held interests such as electronics or trains). Thus, this study was an important step in modifying reward paradigms to include stimuli that are known to be visually salient and behaviorally rewarding for individuals with ASD [45,46]. Results revealed decreased BOLD response in reward regions in response to monetary incentives, corroborating the findings of Scott-Van Zeeland *et al*. [37] and Kohls *et al*. [38]; however, for object images, individuals with ASD showed increased reward system BOLD responses relative to controls. These findings provide support for a model of a 're-directed' neural reward response, that is, a neural response to reward that is intact but responds to different stimuli than in typically developing individuals, rather than a generalized reward deficit in ASD.

The use of a monetary reward as a comparison condition in each of these studies, however, imposes a limitation on their interpretation. Specifically, diminished response to monetary incentives in ASD may reflect generalized, intrinsic differences in neural response to reward, or it may reflect differences in the perceived reward value of money in this population. Monetary reward-specific differences could result from a diminished ability of people with ASD to attribute value to an abstract symbolic representation [43] or even a lack of financial autonomy [41,42] that could impact the perceived value of monetary winnings. In the current study, response to primary reward (food) cues is investigated to address this potential confound and provide more clarity on the responsiveness of the reward system in ASD to nonsocial cues known to be rewarding in typical adults. Because monetary reward studies have shown relative sparing of nonsocial reward compared to social reward and because reduced response in these paradigms may at least partially reflect other cognitive or economic factors, we hypothesized that individuals with ASD would show similar patterns of BOLD response in brain reward regions to a comparison group of typically developing controls in response to images of palatable foods, reflecting intact reward processing for a nonsocial primary reward.

## Methods

### Participants

Nineteen children and adolescents with a diagnosis of ASD and 23 typically developing (TD) controls group-matched for age and gender were recruited for the study through the Vanderbilt Kennedy Center Treatment and Research Institute for Autism Spectrum Disorders (TRIAD) and community advertisements. Cognitive ability was measured for all participants using the Wechsler Abbreviated Scale of Intelligence (WASI [47]); a full-scale IQ score, comprising all four subtests, of at least 70 was required for inclusion in the study. To verify the diagnosis of ASD, individuals in the ASD group were administered the Autism Diagnostic Observation Schedule (ADOS [48]) and parents were interviewed with the Autism Diagnostic Interview-Revised (ADI-R, [49]); both assessments were given by a research-reliable assessor. All children in the ASD group scored above the autism spectrum cutoff on the ADOS, the autism cutoff on the ADI-R, and met Diagnostic and Statistical Manual of Mental Disorders, Fourth Edition (DSM-IV) criteria for ASD based on the judgment of a licensed clinical psychologist. Exclusion criteria included: 1) current use of psychotropic medications (children taking short-acting stimulants (n = 4 in the ASD group) were included but abstained from medication for at least 24 hours to ensure clearance [50]), 2) history of medical conditions associated with autism such as Fragile X, tuberous sclerosis, and epilepsy, recent history of psychiatric or neurologic diagnoses other than ASD, 3) MRI contraindications, and, 4) for the control group, presence of a first degree relative with an ASD. Following these exclusions, further exclusions were made based on post-scan memory task performance and motion artifact (see "Post-scan memory test," and "Preprocessing and quality assurance"). Final sample characterisitics are summarized in Table 1. All parents gave informed consent and participants gave informed assent prior to beginning the first session of the study.

**Table 1:** Participant characteristics of the final included sample, described as mean and (standard deviation)

Group	ADI-R	ADOS	Age	% Male	WASI FSIQ	BMI Percentile
ASD(n = 17)	A: 19.8 (5.57)B:	A: 8.76 (2.01)B:	12.76(2.46)	100%	111.56(12.99)	67.7(31.3)
	15.53 (3.65)C:	3.411 (1.06)C:				
	6.35 (2.44)	1.88 (1.54)				
TD(n = 18)	NA	NA	13.22(3.40)	94%	103.72(13.22)	63.6(23.1)
			t (33) = -.457***P *= **.65	χ^2^(1) = .972*P *= .32	t(33) = 1.75***P *= **.09	t(26) = .397***P *= **.70

ADI-R, combined score on the Autism Diagnostic Interview-Revised; ADOS, combined score on the Autism Diagnostic Observation Schedule. (A: Reciprocal Social Interaction total score, B: Communication total score, C: Restricted, Repetitive, or Stereotyped Patterns of Behavior total score); WASI, Wechsler Abbreviated Scales of Intelligence; BMI, Body mass index (weight in pounds*703/height in inches^2^), given as percentile for age

### Parent report questionnaires

During phone screening, parents were interviewed informally about their child's food preferences and completed the Sensory Profile [51], which includes items targeting food preferences and eating habits (see Additional file 1). For each item on the Sensory Profile, parents rate the question as describing their child's behavior on a scale of 1 to 5, with 1 representing 'Always' and 5 representing 'Never'. While the parents of the ASD group endorsed items related to food pickiness and preferred foods as 'Frequently' or 'Always' more often than controls, children in both groups were screened for food aversions that would be likely to impact their hedonic response to the stimulus set described below. Most examples that parents gave for preferred foods (for example, chicken nuggets, cookies, and so on) were included in the stimulus set.

### fMRI task

#### Block design

**Figure 1:**
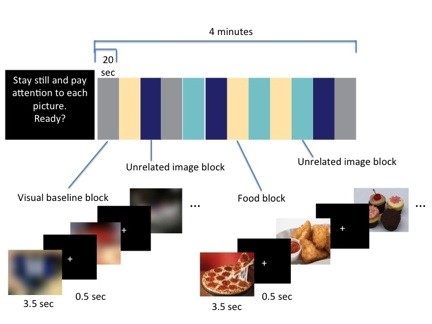
**Block design representing one of the five runs of the fMRI paradigm**. Each run began with an instruction screen, and lasted for 4 minutes, comprised of 12 blocks of 20 seconds each. There were three blocks of food images, three blocks of visual baseline (blurred) images, and three blocks of each of two unrelated conditions, in pseudo-random order (the order of blocks in the run pictured was one of five pseudo-random sequences). Each run always began and ended with the visual baseline conditions. Within each 20-second block, 5 pictures were displayed for 3.5 seconds each, with a 0.5 second black fixation screen to separate the images

Children passively viewed images under conditions of mild fasting (at least four hours without food before the scan). Five four-minute runs were presented during which children viewed images in 20-second blocks (Figure 1). A black screen preceded each run and displayed instructions in white text that indicated the participant should remain still and pay attention to each picture. Participants were told they would be tested after the scan on how well they remembered the pictures. Each block consisted of five images, each presented for 3.5 seconds, followed by a white fixation cross on a black background for 500 milliseconds. Each run had three blocks of each of four conditions, thus 15 images were presented in each of the five runs. In 'food' blocks, the images depicted palatable foods for children (for example, pizza, French fries, ice cream, and so on). In 'baseline' blocks, images from the experimental conditions were rotated 180 degrees and subjected to a Gaussian blur in Photoshop ^® ^(Adobe, San Jose, CA). Two other block types were included during the experiment but were unrelated to the current analysis. Each condition had 38 images, each of which was displayed, using Eprime 2.0 (Psychology Software Tools, Inc., Sharpsburg, PA), twice in randomized order across the five runs, projected onto a screen behind the scanner bore that participants viewed with a mirror attached to the head coil.

#### Image acquisition

All images were acquired using a 3.0 Tesla Philips Achieva MRI scanner with an eight-channel SENSE head coil. Whole-brain functional images were acquired using axial oblique slices (tilted 15° anterior higher than posterior relative to the AC-PC line) with an isotropic 2.5 mm^3 ^voxel size (TR = 2 s, TE = 25 msec, flip angle = 90°, acquisition matrix = 96 × 96, no gap). The first two volumes of each functional run were discarded for equilibration. High-resolution anatomical images were acquired in the sagittal plane using a T1-weighted volumetric 3D SPGR sequence (TR = 7.9 msec, TE = 3.7 msec, flip angle = 7°, acquisition matrix: 256 × 256, 1 mm^3 ^isotropic resolution). Participants were lying comfortably on the scanner bed with foam cushioning between their head and the birdcage coil. During the structural, scout, and reference scans, participants watched a favorite video. During the functional scan, instructions were simply to pay attention to each picture, with the knowledge that they would be tested after the scan to see how many pictures they remembered.

#### Post-scan memory test

Participants were tested after the scanning session to confirm that they were attending during the passive viewing paradigm. The 38 previously viewed food images were combined with 19 novel images and presented in randomized order using Eprime 2.0. Participants were instructed to press '1' on the keyboard if they had seen the image in the scanner, and '2' if they had never seen it before. On each trial, participants were given feedback regarding the accuracy of their response. Hit and false alarm rates were calculated and Z-scored to compute d prime; functional magnetic resonance imaging (fMRI) data for children whose value of d prime was lower than 1.35 (a value that corresponds to a 75% correct rate for both old and new images) were excluded from imaging analysis. Using this criterion, imaging data from one child with ASD and two children with TD were excluded.

### Image processing and analysis

#### Preprocessing and quality assurance

Images were analyzed using SPM5 running in Matlab 7.4.0 (R2007a) (http://www.fil.ion.ucl.ac.uk/spm/). Functional images in each run were realigned to the first volume and resliced. Next, all realigned functional volumes were warped to the standard Montreal Neurological Institute (MNI) template brain for group comparison. Normalized functional images were then smoothed with a Gaussian kernel of 6 mm FWHM.

Realignment parameters were used to identify runs that had >3 mm translation and/or 3° rotation for exclusion from first-level contrast specification. Inclusion of individual participant data in second level analyses required that three or more functional runs met inclusion criteria for first-level analysis. Based on this criterion, three participants from each group were excluded from second-level analysis.

Thus, between exclusions made for poor performance on the post-scan memory task and excess motion, four participants with ASD and five TD participants were excluded, yielding a final sample of 17 in the ASD group and 18 in the TD group. Independent samples t tests confirmed that the final groups did not differ in age (t(33) = -.45, *P *= .65), IQ (t(32) = 1.87, *P *= .07), mean number of included runs (t(33) = 0.25, *P *= .81), or body mass index percentile (t(26) = 0.397, *P *= .70).

#### Statistical analysis

First level analysis was specified for each participant using the general linear model design matrix, modeled using the canonical hemodynamic response function (HRF). The robust weighted least squares (rWLS, [52]) toolbox was used to inversely weight volumes according to their variance due to noise, thereby minimizing the contribution of volumes with motion spikes to the model. Each model was then estimated with the classical restricted maximum likelihood approach for spatially smoothed images. The contrast of interest was defined for each participant by subtracting the baseline from the food condition.

Second level (group) analysis was completed in two stages: 1) using one-sample t tests to create contrasts between conditions within groups, and 2) using two-sample t tests to compare contrasts between the two groups. A region of interest (ROI) mask comprising regions involved in the neural response to rewarding stimuli was created using a combination of automated anatomical labeling (AAL) regions from the Wake Forest University pick atlas [53] for the amygdala, orbitofrontal cortex, anterior cingulate cortex, and insula and the Harvard-Oxford atlas for the nucleus accumbens (http://www.fmrib.ox.ac.uk/fsl/data/atlas-descriptions.html), and applied for all group results. We used a threshold of Z >2.5 (uncorrected *P *< 0.005) and a cluster size of at least ten voxels [54] to identify voxels with a statistically significant BOLD response. We created within-groups contrast maps first to compare activation in the food condition to the visual baseline condition in the ASD and control groups separately. We then created between-group contrast maps that examined the group differences in the activation contrasts specified in the previous step. Significant clusters were localized by converting the MNI coordinates to Talairach coordinates using the Matlab function mni2tal [55] and querying the Talairach coordinates using the Talairach atlas client [56] along with the Talairach and Tournoux atlas [57] for confirmation of anatomical location. Additionally, a whole-brain analysis using a false discovery rate (FDR) corrected threshold of *P *= .05 was performed.

#### Extraction of percent signal change and correlation with ADI-R and Sensory Profile

Functionally-defined regions of interest were created using significant clusters from the group maps (with the exclusion of one cluster that was near the extent threshold, see results) and querying mean percent signal change for each participant within the cluster using Marsbar [58]. These values were used in bivariate correlations with summary scores from the ADI-R algorithm (social, communication, and repetitive behavior subscales), for the ASD group, and Sensory Profile scores that reflect eating habits and food preferences for both groups. Three scores derived from the Sensory Profile were used: a composite score comprising all seven food-related items, and two composite scores that separated these items into food avoiding (four items) and food craving/seeking (three items) categories. Because the distributions of the Sensory Profile scores were skewed, nonparametric correlations (Spearman's rho) were performed.

## Results

### Post-scan memory task

Among those who met inclusion criteria on the post-scan memory task, there was no significant group difference in performance on the task (mean D' for ASD group: 3.74, for TD group: 4.53, t(31) = -1.17, *P *= .25).

### Within-group contrasts

Within the ASD group, the contrast of food images minus blurred baseline visual stimulation yielded significant clusters that exceeeded the extent and intensity thresholds described above in the right anterior and posterior insula, right orbitofrontal cortex, left nucleus accumbens, and bilateral amygdala.

**Figure 2:**
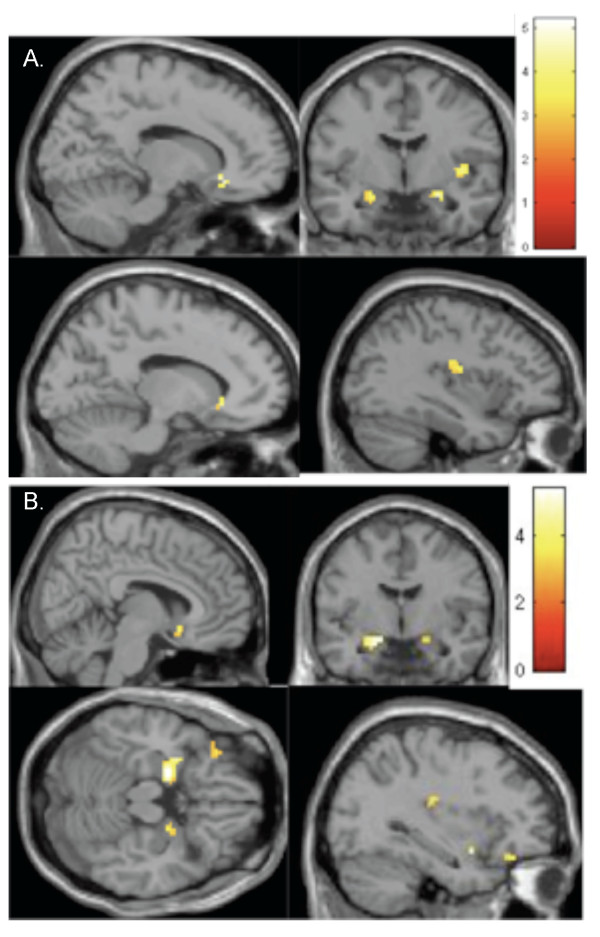
**One sample maps of increased BOLD response to food images in A) the ASD group, and B) the TD group**. (threshold: Z > 2.5, cluster size >10 voxels, *P *< 0.005 (uncorrected)). ASD, autism spectrum disorder; BOLD, blood oxygenation level dependent; TD, typically developing

Within the TD group, this contrast yielded significant clusters of increased BOLD signal in the left orbitofrontal cortex, posterior insula, right nucleus accumbens and bilateral amygdala, with the signal in the right amygdala extending into the ventral temporal cortex. The coordinates and spatial extent of these clusters for the one-sample tests are given in Tables 2 and 3. Figure 2 depicts the maps for within-group contrasts.

**Table 2:** Clusters with significant increases in BOLD signal in the ASD group when contrasting food images with the blurred visual baseline control condition

Region	# voxels	x	Y	z	Z_max_	uncorr *P*
L Amygdala	21	-23	-8	-15	3.38	<0.001
R Amygdala	22	25	-5	-18	3.73	<0.001
L Nucleus accumbens	12	-13	25	-8	3.4	<0.001
L Posterior insula	22	-38	-15	23	3.1	<0.001
R Posterior insula	53	43	-5	3	3.39	<0.001
R Anterior insula	-	45	10	-13	3.33	0.001
Claustrum	-	40	3	-13	3.09	<0.001
R Orbitofrontal cortex	18	15	28	-13	3.93	0.001

ASD, autism spectrum disorder; BOLD, blood oxygenation level dependent; L, left; R, right; uncorr, uncorrected

**Table 3:** Clusters with significant increases in BOLD signal in the TD comparison group when contrasting food images with the blurred visual baseline control condition

Region	# voxels	x	y	z	Z_max_	uncorr *P*
L Amygdala	94	-20	-3	-20	4.09	< 0.001
	-	-30	3	-18	3.52	< 0.001
R Amygdala/ventral temporal	33	25	3	-28	3.44	< 0.001
	-	23	-5	-18	3.02	0.001
R Nucleus Accumbens	12	8	15	-13	4.2	< 0.001
L Posterior insula	10	-33	-23	23	2.98	0.001
	-	-30	-25	15	2.84	0.002
L Orbitofrontal cortex	21	-35	33	-20	3.38	< 0.001

Spatial coordinates are in MNI space

BOLD, blood oxygenation level dependent; L, left; MNI, Montreal Neurological

Institute; R, right; TD, typically developing; uncorr, uncorrected

### Between-group contrasts

**Figure 3:**
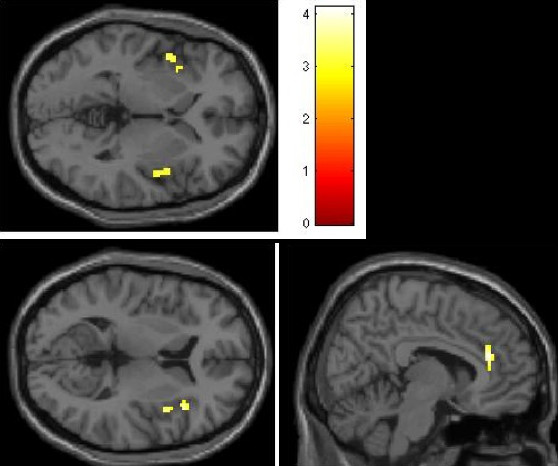
**Regions (insula and anterior cingulate cortex) showing increased neural response to food cues in ASD compared to TD children (threshold: Z >2.5, cluster size >10 voxels, *P *< 0.005 (uncorrected))**. No activations within the regions of interest met this threshold in the reverse (TD > ASD) contrast. ASD, autism spectrum disorder; TD, typically developing; uncorr, uncorrected

When subtracting the BOLD signal for food - baseline contrast between groups, the contrast in which the ASD group had a higher signal than the TD group included clusters in the bilateral insula and anterior cingulate cortex, whereas the reverse contrast yielded no significant clusters. The results of these contrasts are described in Table 4 and Figure 3. The results of the whole brain analysis revealed no significant activation in one group relative to the other at the FDR-corrected threshold of *P *= .05.

**Table 4:** Clusters with significantly more BOLD signal in the ASD group versus the TD comparison group when contrasting food images with the blurred visual baseline control condition

	# voxels	x	y	z	Z_max_	uncorr *P*
ASD > Control						
R Posterior insula	48	48	-3	3	3.54	<0.001
	-	43	5	5	3.48	<0.001
R Anterior insula	11	40	20	8	3.34	<0.001
L Mid insula	23	-45	5	3	3.19	0.001
		-38	13	-3	2.83	0.002
R Anterior cingulate cortex	25	8	35	20	3.69	<0.001
Control > ASD (No significant clusters)						

Spatial coordinates are in MNI space. The reverse contrast, TD > ASD, yielded no significant clusters at this threshold. ASD, autism spectrum disorder; BOLD, blood oxygenation level dependent; L, left; MNI, Montreal Neurological Institute; R, right; TD, typically developing; uncorr, uncorrected

### Correlation of percent signal change with questionnaire variables

There were no significant correlations between percent signal change in the functionally defined ROIs (insula and cingulate) and any of the algorithm summary scores on the ADI-R for the ASD group. For both groups combined, the mean BOLD percent signal change within the largest cluster (the right posterior insula (48 voxels surrounding 48-3 3) showed a trend-level positive correlation with the Sensory Profile composite variable indexing unusual eating habits or food preferences generally (*P *= .321, *P *= .060). When this composite variable was separated into subcomponents, the percent signal change was found to correlate both with the component indexing food cravings or positive preferences (*P *= .323, *P *= .058) and the component indexing food aversions or pickiness (*P *= .256, *P *= .137). When correlations were performed separately by group, none approached statistical significance.

## Discussion

Little is known about the neural basis of response to primary reward in ASD. As a first step, we probed the reward system using images of appetizing foods for children under conditions of mild fasting, a paradigm which has been demonstrated previously to recruit neural reward networks [32,59]. Our findings demonstrate that neural reward system response to food cues is not only intact, but may even be enhanced in children with ASD. This was found despite the well-known elevation of food selectivity in children with ASD [60-62] and diminished gustatory discrimination ability in ASD [63]. The foods we chose to depict were specifically targeted to be palatable to children, and were exclusively high-calorie foods, with images representing both sweet and savory flavors. High calorie foods have been demonstrated to be potent activators of neural reward circuitry [64,65]. Foods that were represented heavily in this stimulus set (for example, starchy foods, chicken nuggets, chocolate, pizza) were consistent with parent reports of food preferences for children in our sample, supporting the notion that our food images were appealing to children across groups.

Although both groups showed increased BOLD response to food images in a similar network of regions known to mediate reward, when comparing the ASD and TD groups directly, we found greater response in the ASD group in the insula and anterior cingulate cortex (ACC), known for their roles in assessing interoceptive states [15,66], and evaluating and preparing for response based on the motivational significance of these states [67], respectively. These two regions are frequently co-activated in fMRI studies, and have been found to constitute a resting state network (the 'salience' network) [68,69]. The ACC has been demonstrated to be hyperactive in previous neuroimaging studies of reward in ASD [40,44]. The degree of connectivity between the insula and ACC at rest has been shown to be related to autistic traits in the general population [70].

The insula and ACC have been postulated by Craig [71,72] to constitute an integrated system of emotional perception and action, analagous to primary sensory and motor cortices. Included in Craig's model is the unique concentration of von Economo neurons in these two regions, which he proposes form the basis for rapid communication between them despite their physical separation. A recent neuroanatomical study reported a higher proportion of Von Economo neurons to pyramidal neurons in the insulae of their sample with ASD relative to controls [73] and the authors theorized that this neural difference might give rise to heightened interoception. Our result of enhanced response in insula and ACC to food cues in ASD may thus suggest that children in the ASD group were more attuned to an internal state of hunger or food craving elicited by the pictures than controls.

The work of Craig and others has demonstrated a posterior-anterior gradient of interoceptive representation within the insula, with posterior regions responding to objective features of the stimuli themselves and more anterior regions to more subjective assessment of their emotional significance [74,75]. It is of note that our comparison of the ASD > TD contrast revealed three distinct clusters of significantly higher response in the insulae of the ASD group, distributed along this axis (Figure 1b). This suggests that they may have experienced both stronger signals of hunger or 'wanting' the food in the images, as well as a more intense emotional reaction to these interoceptive signals. The role of the insula in integrating interoceptive sensation with reward evaluation in the context of reward-motivated behavior such as drug craving is currently being actively investigated [76,77].

The insula is responsive to visual food cues [29,78] and is also the site of the primary gustatory cortex, although recent studies provide evidence that a more accurate characterization is a multimodal oral sensory region that integrates taste with other sensory features such as texture and temperature [79]. While the primary taste cortex occupies the most anterior region of the insula in nonhuman primates [80,81], it is positioned further posterior in humans [82]. The most anterior portion of the human insula has been hypothesized to have evolved more recently along with increased human capacity for self-awareness [71,72]. Although not statistically significant, the positive correlation of the BOLD response in the insula with parent reports of food cravings and preferences is consistent with the known function of this region. Further work is needed to explore the differences in insula response in ASD exhibited in the current study. The lack of significant correlation between BOLD response in these regions and ADI-R scores summarizing clinical severity of ASD may suggest that the enhanced response in these regions is unrelated to core features of ASD, or it may reflect a lack of power to detect a relationship, possibly due to small sample size and/or the diagnostic rather than quantitative nature of the ADI-R algorithm.

## Conclusion

Despite an aberrantly enhanced response in the insula and anterior cingulate in the ASD group, the orbitofrontal cortex, nucleus accumbens, and amygdala were similarly responsive in both groups, although we noted slight differences in the laterality of response in the nucleus accumbens and OFC. Thus, all nodes in the neural reward circuit are responsive to primary reward in ASD, suggesting that social deficits are not explainable by a generalized under-responsiveness of the reward system.

This study is a first step in assessing neural response to primary rewards in ASD although much more work needs to be done to fill in remaining gaps. Although children in both groups fasted for the same minimum amount of time, subjective hunger ratings and/or hedonic ratings of food images would be an important variable for future studies to collect and report. Further, our paradigm did not allow us to separate motivational from hedonic aspects of food reward. Additional fMRI studies incorporating an anticipatory phase and actual palatable food delivery, or utilizing behavioral paradigms that confer the ability to separate 'liking' from 'wanting' (for example, [83]), should be undertaken in the future. An important next step will also be to directly compare food reward with social and object reward cues, to provide a clearer picture of the reward system as a whole in ASD. Finally, application of neuroimaging and reward paradigms to younger children and/or at-risk sibling groups will facilitate the translation of this knowledge into new approaches for early identification and intervention in ASD. The current finding of enhanced response to primary reward advances our understanding of the similarities and differences in the brain's response to rewarding stimuli in ASD; this understanding will ultimately provide opportunities to harness the power of the reward system to optimize educational and treatment approaches in children with ASD.

## Abbreviations

AAL: Automated anatomical labeling; ACC: Anterior cingulate cortex; ADI-R: Autism Diagnostic Interview, Revised; ADOS: Autism Diagnostic Observation Schedule; ASD: Autism spectrum disorder; BMI: Body mass index; BOLD: Blood oxygenation level-dependent; DSM-IV: Diagnostic and Statistical Manual of Mental Disorders, 4^th ^Edition; MRI: Magnetic resonance imaging; NAc: Nucleus accumbens; OFC: Orbitofrontal cortex; ROI: Region of interest; VMPFC: Ventromedial prefrontal cortex; VTA: Ventral tegmental area; WASI: Wechsler Abbreviated Scales of Intelligence

## Competing interests

The authors declare that they have no competing interests.

## Authors' contributions

JF assisted with image analysis, participant recruitment and assessment, and helped to draft the manuscript. JH coordinated participant recruitment, scheduling, and scan acquisition. CN performed clinical assessments and provided expert clinical opinion for inclusion of participants in the ASD group. BR, MB and RC assisted with study design and interpretation. BR and AC assisted with image processing and analyses. CC conceived of the study, participated in its design, coordination, and analyses, and drafted the manuscript. All authors read and approved the final manuscript.

## Supplementary Material

Additional file 1 **Relevant Items from the Sensory Profile (Dunn, 1999) used to assess food preferences and aversions**. These items were ranked on a scale of 1 ("always") to 5 ("never"). Items listed for questions 56, 57, 61, 62, and 63 were used to optimize the stimulus set. Click here for file
