# Supplementary material for: Response of neural reward regions to food cues in autism spectrum disorders
Source: J Neurodev Disord. 2012 May 17;4(1):9. doi: 10.1186/1866-1955-4-9 (PMC3436657; doi:10.1186/1866-1955-4-9)
Supplement: Additional file 1 — Relevant Items from the Sensory Profile (Dunn, 1999) used to assess food preferences and aversions. These items were ranked on a scale of 1 ("always") to 5 ("never"). Items listed for questions 56, 57, 61, 62, and 63 were used to optimize the stimulus set. [file 1866-1955-4-9-S1.DOC]

Appendix A

Relevant Items from the Sensory Profile (Dunn, 1999) used to assess food preferences and aversions. These items were ranked on a scale of 1 (“always”) to 5 (“never”). Items listed for questions 56, 57, 61, 62, and 63 were used to optimize the stimulus set.

55. Avoids certain tastes or food smells that are typically part of children’s diets

56. Will only eat certain foods (list:)

57. Limits self to particular food textures/temperatures (list:)

58. Picky eater, especially regarding food textures

61. Shows strong preference for certain tastes (list:)

62. Craves certain foods (list:)

63. Seeks out certain tastes or smells (list:)
